# Radioactivity of Drinking-Water in the Vicinity of Nuclear Power Plants in China Based on a Large-Scale Monitoring Study

**DOI:** 10.3390/ijerph10126863

**Published:** 2013-12-06

**Authors:** Xiao-Xiang Miao, Yan-Qin Ji, Xian-Zhang Shao, Huan Wang, Quan-Fu Sun, Xu Su

**Affiliations:** China CDC Key Laboratory of Radiological Protection and Nuclear Emergency, National Institute for Radiological Protection, Chinese Center for Disease Control and Prevention, Beijing 100088, China; E-Mails: miaoxxnirp@gmail.com (X.-X.M.); shaoxzh@nirp.cn (X.-Z.S.); cdcwanghuan@aliyun.com (H.W.); qfusun@nirp.cn (Q.-F.S.); suxu@nirp.cn (X.S.)

**Keywords:** radioactivity, gross α and gross β, drinking-water, vicinity, nuclear power plant, monitoring

## Abstract

The public concern for radioactivity of drinking-water has been increasing in recent years after the rapid development of nuclear power plants, and especially after the Fukushima nuclear accident. In this study, the radioactivity of water samples collected in the vicinity of nuclear facilities from seven provinces in China was measured and an average annual equivalent effective dose derived from drinking-water ingestion was calculated. The results showed that, in winter and spring, the activities of gross α and β ranged from 0.009 Bq/L to 0.200 Bq/L and from 0.067 Bq/L to 0.320 Bq/L, respectively. While, in summer and autumn, the activities of gross α and β varied from 0.002 Bq/L to 0.175 Bq/L and from 0.060 Bq/L to 0.334 Bq/L. Our results indicated that the gross α and β activities in these measured water samples were below the WHO recommended values (0.5 Bq/L for gross α and 1.0 Bq/L for gross β) and the annual equivalent effective dose derived from drinking-water ingestion was at a safe level.

## 1. Introduction

Drinking-water is one of the most essential and indispensable substances for human life or actually for the whole living world [1]. The safety of drinking-water is often of the highest priority for public health and environmental protection and meanwhile to access sustainable safe drinking-water had become one of the United Nations Millennium Development Goals [2]. Because of the occurrence of natural radionuclides, radioactivity of drinking-water is always an important judging criterion for the quality of drinking-water, just as what microbiological and chemical criteria are [3,4]. Generally, radioactivity of drinking-water means the sum of gross α and gross β activity. Gross α activity is the total activity of all α emitters such as ^210^Po, ^226^Ra, ^238^U, when radon has been eliminated. While gross β activity is the total activity of all β emitters excluding tritium, ^14^C and other weak β emitters [5].

According to the report of United Nations Scientific Committee on the Effects of Atomic Radiation (UNSCEAR) in 2000, radiation exposure from consumption of drinking-water and food contributes about 8% of the total natural radiation exposure for humans, which includes external and internal sources of radiation [5,6]. The guidelines for assessing the safety of drinking-water have provided by World Health Organization (WHO), International Commission on Radiological Protection (ICRP) and many other national regulations. As the most common standard, guideline values for drinking-water recommended by WHO are 0.5 Bq/L for gross α activity and 1.0 Bq/L for gross β activity so as to guarantee an exposure lower than 0.1 mSv·y^−1^ [5]. 

From the beginning of nuclear power development in the last century, more than 500 nuclear power reactors have been or being built in the World, and in China, so far, there are about 45 nuclear power reactors which are operating or are under construction [7]. Meanwhile, public attention paid to the safety of drinking-water in the vicinity of nuclear power plants has been growing these years, especially after the Fukushima nuclear accident in 2011. There were also some emergency monitoring reports concerning this accident in China [8,9]. However, there are limited reports involving the radioactivity of drinking-waters surrounding the nuclear facility in China, specifically there is a shortage of documents about large scale testing programs. The focus of this study was therefore to evaluate the radioactivity, mainly gross α and β activity, in the vicinity of eight nuclear power plants in seven provinces of China and to establish radioactive background baselines for these areas.

## 2. Experimental Section

### 2.1. Study Area

**Figure 1:**
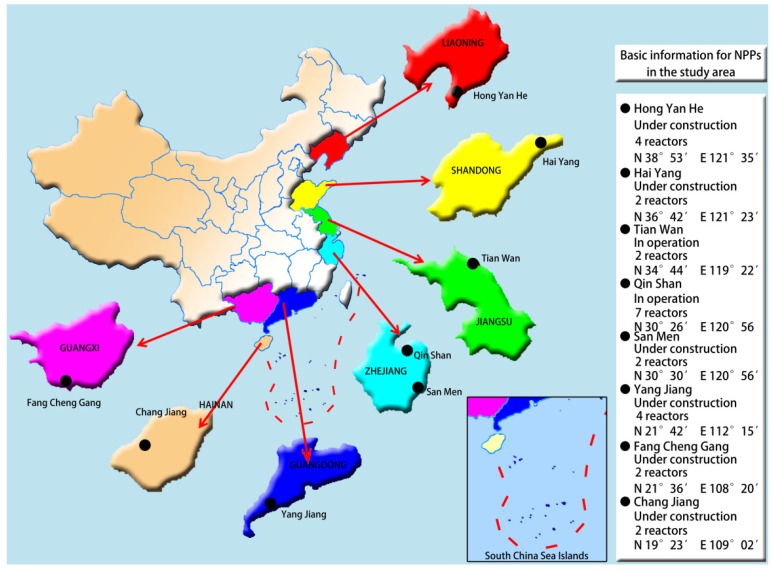
Basic information for Nuclear Power Plants (NPPs) in the study area.

In order to evaluate the influences of radioactivity in drinking-water and to establish the radioactive background baselines of water samples around the nuclear power plants, eight nuclear facilities which are operating or under construction and located in seven provinces of China (Figure 1) were selected for this investigation.

### 2.2. Sample Collection and Preparation

All of the water samples were collected from different locations within 30 km around the nuclear power plants. Water samples were separated two groups. One was collected in winter and spring (also called dry season) and the other in summer and autumn (called wet season). According to the different source, water samples were named tap water, well water and others. Tap water originated from surface water such as rivers and reservoirs and well water was derived from ground water. Besides, spring water and stream water were attributed as others.

The collection and preservation of water samples were performed following Chinese national standard [10] and WHO guidelines [5]. All of the samples were collected in pre-cleaned polyethene containers and then acidified with dilute of nitric acid to a final concentration of 2% for minimizing precipitation and absorption of particulates in the water on containers walls. Before analysis, one liter of acidified water was added in a beaker and heated to sub-boiling on electric heating plate. The water samples were transferred to an evaporating dish when the volume of water was less than 50 mL. Then one milliliter concentrated sulfuric acid was added in the water samples and the samples were heated until the residue produced. Finally the residues were added into a muffle furnace at 350 °C for an hour. 

### 2.3. Sample Analysis

The radioactivity of gross α and β was measured by using α/β counting system [11,12]. The α/β counters of the low background multiple detector with different models were used as counting instruments and detailed information was shown in Table 1.

**Table 1:** Instrument information for gross α and gross β counting system in each province.

Number	Province	Instrument Model	Source for α Calibration	Source for β Calibration	Active Area Diameter (mm)
1	Liaoning	BH-1216	^241^Am/Uranium	^4^^0^K	20
2	Jiangsu	MPC-9604	^241^Am	^9^^0^Sr/^9^^0^Y	30.25
3	Zhejiang	BH-1216	^241^Am/Uranium	^4^^0^K	25
4	Shandong	BH-1216II	^241^Am	^4^^0^K	20
5	Guangdong	MPC-9604	^241^Am	^9^^0^Sr/^9^^0^Y	20
6	Guangxi	MPC-9604	^241^Am	^4^^0^K	29
7	Hainan	LB-2008	^239^Pu	^9^^0^Sr/^9^^0^Y	25

### 2.4. Quality Control

To ensure the accurate and reliable determined results in the seven provinces, first, all the instruments involving in examining the samples were verified by the national metrology department; second, all the laboratories of the participating provinces had performed the quality control intercomparison analysis.

## 3. Results and Discussion

### 3.1. Gross α and Gross β Activity of Drinking-Water for Each Monitoring Province in Dry and Wet Season

The gross α activities determined from all of water samples collected in the dry season ranged from 0.009 ± 0.008 Bq/L (Guangxi) to 0.200 ± 0.127 Bq/L (Shandong) while, the gross β activities ranged from 0.067 ± 0.065 Bq/L (Guangxi) or 0.067 ± 0.068 Bq/L (Haninan) to 0.320 ± 0.317 Bq/L (Shandong) (Table 2). The measured gross α and gross β activities in all water samples were below the WHO recommended maximum contamination values (0.5 Bq/L for gross α, 1.0 Bq/L for gross β). According to the determined gross α activity, the descending order of the seven provinces was Shandong > Hainan > Jiangsu > Liaoning > Guangdong > Zhejiang > Guangxi. While the gross β order was Shandong > Guangdong > Zhejiang > Jiangsu > Liaoning > Guangxi = Hainan. The radioactivity of water is usually related to the geological characteristics of the soils. For instance, the radioactive elements could be retained in sandstone and igneous rocks more easily and enter the environment, including water, through leaching processes [13] and the radioactivity levels at sites around uranium ore deposits are generally higher than at other sites [14]. Further investigations need to address the correlation between water radioactivity and the geological characteristics of soils in these sampling sites.

For the wet season, because Guangdong Province did not successfully complete the sample collection, the gross α and β radioacitivies in water samples were analysed for the other six provinces (Table 2). The mean gross α activity was from 0.002 ± 0.004 Bq/L (Hainan) to 0.175 ± 0.127 Bq/L (Shandong), while the mean gross β activity varied from 0.060 ± 0.041 Bq/L (Hainan) to 0.334 ± 0.273 Bq/L (Liaoning). Like with the dry season, the result of Shandong Province gave the highest gross α value while the gross α and β results were all under the WHO recommended maximum contamination values and recommended values, respectively. The descending order for the six monitored provinces was Shandong > Liaoning > Jiangsu > Zhejiang > Guangxi > Hainan. For gross β activity, the order was Liaoning > Shandong > Zhejiang > Jiangsu > Guangxi > Hainan. It could be seen that the water radioactivity for Hainan Province was still the lowest one. On the other hand, for Shandong Province, the gross β activity of water was lower than the activity of Liaoning Province, but the total radioactivity still seemed high in the sampling sites. Our results indicated that the radioactivity of water samples changed following the different seasons, particularly in Hainan and Liaoning provinces. 

**Table 2:** Range, mean value with standard deviation of gross α and gross β activity for each monitoring province in dry and wet season (Bq/L).

			Liaoning	Jiangsu	Zhejiang	Shandong	Guangdong	Guangxi	Hainan
Dry season	Gross α	*n*	5	10	11	5	14	15	23
		Range	0.020–0.220	ND–0.110	ND–0.053	ND–0.289	ND–0.100	ND–0.027	ND–0.334
		Mean	0.074	0.078	0.041	0.200	0.053	0.009	0.109
		S.D	0.083	0.018	0.015	0.127	0.029	0.008	0.131
	Gross β	*n*	5	10	11	5	14	15	23
		Range	0.040–0.150	ND–0.140	0.06–0.256	0.045–0.859	0.070–0.420	0.003–0.280	0.012–0.236
		Mean	0.080	0.114	0.137	0.320	0.144	0.067	0.067
		S.D	0.045	0.023	0.072	0.317	0.084	0.065	0.068
Wet season	Gross α	*n*	5	10	10	6	*	15	24
		Range	0.050–0.280	0.050–0.120	ND–0.027	0.048–0.412	*	ND–0.005	ND–0.012
		Mean	0.124	0.076	0.024	0.175	*	0.013	0.002
		S.D	0.090	0.024	0.004	0.127	*	0.010	0.004
	Gross β	*n*	5	10	10	6	*	15	24
		Range	0.070–0.770	0.050–0.140	0.076–0.328	ND–0.630	*	0.014–0.220	0.031–0.232
		Mean	0.334	0.100	0.144	0.257	*	0.070	0.060
		S.D	0.273	0.028	0.100	0.227	*	0.076	0.041

ND: Non detectable; * No corresponding data.

### 3.2. Gross α and Gross β Activity of Drinking-Water for Different Water Types in Each Monitoring Province

The measured gross α and gross β activities of different sources of drinking-water from the seven provinces were given in Table 3. The gross α and β radioactivities detected in tap waters showed that the samples from Shandong Province gave the highest values of gross α (0.169 ± 0.131 Bq/L) and gross β (0.327 ± 0.276 Bq/L), which slightly exceeded the WHO limit value (0.1 Bq/L for gross α) and were also under the maximum contamination limit (0.5 Bq/L for gross α). Considering well water, since Shandong and Guangdong provinces did not provide values for well water samples, the ranges of mean activity of gross α and gross β were from 0.024 ± 0.044 Bq/L (Guangxi) to 0.106 ± 0.088 Bq/L (Liaoning) and from 0.066 ± 0.052 Bq/L (Guangxi) to 0.213 ± 0.241 Bq/L (Liaoning), respectively. For other types of water, due to sampling reason, only four provinces successfully had collected this type of water samples and completed the measurments. The mean gross α and gross β activities were from 0.020 ± 0.001 Bq/L (Zhejiang) to 0.173 ± 0.228 Bq/L (Hainan) and from 0.124 ± 0.015 Bq/L (Guangdong) to 0.234 ± 0.002 Bq/L (Hainan), respectively. These results demonstrated that gross α and β radioactivities in most of the tap and well water samples were below 0.15 Bq/L, indicating that the radioactivities of these sources of drinking-water are at a safe level. Moreover, changes of gross α and β activity were noticed in different locations and seasons, even in different sources of water.

**Table 3:** Gross α and gross β activity for different kind of water samples at each monitoring site.

			Liaoning	Jiangsu	Zhejiang	Shandong	Guangdong	Guangxi	Hainan
Tap water	Gross α	*n*	1	14	11	11	9	24	29
		Range	*	ND–0.110	ND–0.053	ND–0.412	ND–0.100	ND–0.027	ND–0.045
		Mean	0.040	0.075	0.048	0.169	0.063	0.010	0.012
		S.D	*	0.019	0.005	0.131	0.033	0.007	0.018
	Gross β	*n*	1	14	11	11	9	24	29
		Range	*	ND–0.140	0.060–0.328	ND–0.859	0.070–0.420	0.023–0.064	0.012–0.221
		Mean	0.150	0.101	0.168	0.327	0.154	0.045	0.050
		S.D	*	0.028	0.093	0.276	0.105	0.013	0.036
Well water	Gross α	*n*	9	2	2	*	*	6	14
		Range	0.020–0.280	0.050–0.070	ND–0.027	*	*	ND–0.041	ND–0.112
		Mean	0.106	0.060	0.027	*	*	0.022	0.024
		S.D	0.088	0.014	*	*	*	0.016	0.044
	Gross β	*n*	9	2	2	*	*	6	14
		Range	0.040–0.770	0.090–0.120	0.069–0.081	*	*	0.014–0.280	0.031–0.236
		Mean	0.213	0.105	0.075	*	*	0.133	0.066
		S.D	0.241	0.021	0.008	*	*	0.106	0.052
Others	Gross α	*n*	*	4	8	*	5	*	2
		Range	*	ND–0.120	ND–0.021	*	0.020–0.090	*	0.012–0.334
		Mean	*	0.097	0.020	*	0.046	*	0.173
		S.D	*	0.021	0.001	*	0.027	*	0.228
	Gross β	*n*	*	4	8	*	5	*	2
		Range	*	ND–0.140	0.061–0.275	*	0.110–0.140	*	0.232–0.235
		Mean	*	0.127	0.127	*	0.124	*	0.234
		S.D	*	0.015	0.073	*	0.015	*	0.002

ND: Non detectable; * No corresponding data.

### 3.3. Radiation Exposure through Ingestion of Different Types of Drinking-Water in Each Monitoring Province

For humans, exposure from drinking-water is mainly due to naturally occurring radionuclides in the thorium and uranium series. Even though the contribution of drinking-water to total exposure is small, there are also guidelines for this kind of exposure to guard against deleterious radiological health effects. According to the WHO, a reference dose level of committed effective dose, equal to 0.1 mSv year’s ingestion of drinking-water is recommended [5]. 

To assess the health risk on the public in each monitoring province, the annual effective dose (AED) associated with radiation exposure through ingestion of different types of drinking-water was calculated using the following equation:

AED = *A* × *V* × *C* (1)

where *A* is the activity of the gross α ( Bq/L ); *V* is the annual ingested volume of drinking-water for adults (730 L·y^−1^ recommended by WHO) [5]; *C* is the dose coefficient for ingestion by adults (3.58 × 10^−4^ mSv/Bq for the gross α) [15,16]. The AED values for tap water were from 0.005 mSv (Guangxi) to 0.088 mSv (Shandong) and for well water and others, it varied from 0.012 mSv (Guangxi) to 0.055 mSv (Liaoning) and from 0.010 mSv (Zhejiang) to 0.090 mSv (Hainan), respectively (Table 4). These results indicated that the calculated AED values of seven provinces were below the WHO recommended reference value of 0.1 mSv, so the health risk on the public caused by radiation exposure through drinking-water ingestion in these monitoring provinces was within a reasonable scope. The annual ingested volume of drinking-water is dependent on age and area. Because of the shortage of the density function of annual ingested volume of drinking-water in these regions, the recommended volume of drinking-water for adults was employed for the calculation of AED. The calculation of AED with different volumes of drinking-water combining with different ages and areas need to be carried out in future. In general, the radioactivity level of well water is higher compared with tap water [17], however the calculated AEDs of well water from Jiangsu and Zhejiang were less than the AEDs from tap water (Table 4). This maybe due to the different sources of tap water. For example, river water and reservoir water were the main sources of tap water in some provinces. It is sometimes possible that the background radioactivity level of river and reservoir water is higher than that of some well water. Besides, the geological characters of Jiangsu and Zhejiang may have impact on the radioactivity levels of the sources of tap water. It may also be the reason of the distance between the nuclear site and the intake point of water sample. The details of the impacts on the radioactivity levels in tap water need further investigations.

**Table 4:** The annual effective dose calculated for different kind of water samples at each monitoring site (mSv).

	Liaoning	Jiangsu	Zhejiang	Shandong	Guangdong	Guangxi	Hainan
Tap water	0.021	0.039	0.025	0.088	0.033	0.005	0.006
Well water	0.055	0.031	0.014	*	*	0.012	0.013
Others	*	0.051	0.010	*	0.024	*	0.090

* No corresponding data.

### 3.4. Comparison This Work with Others on the Gross α and Gross β Activity of Different Types of Drinking-Water in Different Places

The determined gross α and gross β activity for different types of water in seven provinces of this work was compared with those reported in other works in recent years (Table 5). The data showed that the gross α and β activities were related to different types of water and different places. For example, the maximum values of gross α and β activity exceed the WHO recommended values in Katsina State in Nigeria [18] and the Balaton Upland region of Hungary [19], so it is necessary to investigate the concentrations of individual radionuclides and compare them with specific guidance levels for the abnormal water samples.

**Table 5:** Comparison on the range of gross α and gross β activity among different places.

Place and Country	Type of water	Gross α (Bq/L)	Gross β (Bq/L)	Reference
Seven provinces, China	Tap water	0.010–0.169	0.045–0.327	This work
	Well water	0.022–0.106	0.066–0.213	
	Spring/stream *etc.*	0.020–0.173	0.124–0.234	
Milano, Italy	Tap water	<0.0077–0.349	<0.025–0.273	Rusconi, 2004 [20]
Dhaka city, Bangladesh	Tap water	0.0019–0.0082	0.0293–0.1157	Fedous, 2012 [21]
Eastern Black Sea Region, Turkey	Tap water	0.0002–0.015	0.0252–0.2644	Damla, 2006 [22]
Amman, Jordan	Tap water	<0.05–0.2495	<0.1879–0.3270	Sajedah Al-Amir, 2009 [23]
Katsina State, Nigeria	Well water	0.080–2.300	0.120–4.970	Muhammad, 2010 [18]
Nevşehir province, Turkey	Well water	0.080–0.380	0.120–3.470	Turhan, 2013 [24]
Balaton Upland region, Hungary	Spring water	0.026–1.749	0.033–2.015	Jobbαgy, 2013 [19]
Zacatecas and Guadaluoe, Mexico	Mineral water	<0.011–0.415	<0.026–0.695	Davila Rangel, 2001 [25]
Greece	Bottled water	0.008–0.094	0.071–0.350	Karamanis, 2007 [26]

## 4. Conclusions

In this study, the gross radioactivity of water samples collected in the vicinity of eight nuclear power plants in China was investigated. The results showed here that the gross α and β radioactivities in different water samples were less than 0.5 Bq/L or 1.0 Bq/L, respectively. Moreover the equivalent effective exposure dose for different types of drinking-water derived from water ingestion was estimated and the results indicated that the water in these investigated locations is at a safe level. In addition, our results support the notion that radioactivity of water relates to the location with geological characteristics and can be influenced in different seasons and changes in different sources.

To the best of our knowledge, this is the first large-scale study of gross α and gross β activity concentration in drinking-water samples in the vicinity of the nuclear power plants in China. The results would be useful to set radioactive background baselines for these areas and be helpful for reducing public concerns about radioactivity contamination around nuclear power plants. 
